# Microglia-derived IL-1β promotes chemokine expression by Müller cells and RPE in focal retinal degeneration

**DOI:** 10.1186/s13024-017-0175-y

**Published:** 2017-04-24

**Authors:** Riccardo Natoli, Nilisha Fernando, Michele Madigan, Joshua A. Chu-Tan, Krisztina Valter, Jan Provis, Matt Rutar

**Affiliations:** 1 The John Curtin School of Medical Research, The Australian National University, Canberra, ACT Australia; 2 ANU Medical School, The Australian National University, Canberra, ACT Australia; 3 Save Sight Institute, Discipline of Clinical Ophthalmology, The University of Sydney, Sydney, NSW Australia; 4 School of Optometry and Vision Science, The University of New South Wales, Kensington, NSW Australia; 5 The University of Melbourne, Parkville, VIC Australia

**Keywords:** Retinal degeneration, Microglia, Interleukin-1β, IL-1β, Chemokines, RPE, Müller cells, Macrophages, Age-related macular degeneration, AMD

## Abstract

**Background:**

Chemokine signalling is required for the homing of leukocytes during retinal inflammation, and is associated with pathogenesis of diseases such as age-related macular degeneration (AMD). Here, we explore the role of interleukin-1β (IL-1β) in modulating AMD-associated chemokines *Ccl2, Cxcl1,* and *Cxcl10* during photo-oxidative retinal damage, and the effect on both the accumulation of outer-retinal macrophages, and death of photoreceptors.

**Methods:**

Inhibition of retinal IL-1β expression was performed using either siRNA or antibody neutralisation, which was intravitreally injected in SD rats prior to photo-oxidative damage. Changes in the expression and localisation of *Il-1β*, *Ccl2*, *Cxcl1* and *Cxcl10* genes were assessed using qPCR and *in situ* hybridisation, while the recruitment of retinal macrophages was detected using immunohistochemistry for IBA1. Levels of photoreceptor cell death were determined using TUNEL.

**Results:**

Photo-oxidative damage elevated the expression of *Il-1β* and inflammasome-related genes, and IL-1β protein was detected in microglia infiltrating the outer retina. This was associated with increased expression of *Ccl2, Cxcl1*, and *Cxcl10.* Intravitreal IL-1β inhibitors suppressed chemokine expression following damage and reduced macrophage accumulation and photoreceptor death. Moreover, in Müller and RPE cell cultures, and in vivo, *Ccl2*, *Cxcl1* and *Cxcl10* were variously upregulated when stimulated with IL-1β, with increased macrophage accumulation detected in vivo.

**Conclusions:**

IL-1β is produced by retinal microglia and macrophages and promotes chemokine expression by Müller cells and RPE in retinal degeneration. Targeting IL-1β may prove efficacious in broadly suppressing chemokine-mediated inflammation in retinal dystrophies such as AMD.

## Background

Inflammation plays a key role in the pathogenesis of age-related macular degeneration (AMD), which is the leading cause of blindness in the ageing population of the Western world [1]. One of the characteristics of atrophic or ‘dry’ AMD, is the accumulation of microglia/macrophages in the outer retina and subretinal space [2–6]. Homing of leukocytes, such as macrophages, to sites of neuronal damage is orchestrated in part by the co-ordinated expression of chemokines (reviewed in [7]). The chemokine family consists of numerous ligands and receptors belonging to particular subclasses (such as Ccl- and Cxcl-), which act as guidance cues for leukocytes during homeostasis and injury [8]. Chemokine expression is prominent in many retinal degenerations, including AMD, wherein the up-regulation of genes encoding potent ligands such as *Ccl2* and *Cxcl10* is a characteristic of the disease [9]. The Ccl2-Ccr2 signalling axis has been well-studied in relation to retinal disease, and ablation or pharmacological inhibition of the ligand or receptor exacerbates pathology in laser-induced neovascularisation and photo-oxidative damage models [10–12].

Our previous work has shown that RPE and Müller cells are the mediators of chemokine responses, and up-regulate the expression of *Ccl2*, *Cxcl1* and *Cxcl10* in response to damage [13]. Furthermore, pharmacological suppression of the Ccl- and Cxcl- signalling axes ameliorates subretinal macrophage infiltration and photoreceptor/RPE degeneration [14]. However, the factor/s that stimulate expression of these chemokines during retinal inflammation remain unclear. Recent in vitro studies indicate that cytokines such as *Il-6* and *Ccl2* may be stimulated in RPE or Müller cells when co-cultured with lipopolysaccharide (LPS)-stimulated microglia [15, 16], suggesting that similar interactions may promote chemokine expression by Müller cells and RPE during retinal degeneration*.*

IL-1β is a pro-inflammatory cytokine whose maturation and secretion into the extracellular environment is mediated by assembly of the NLRP3 inflammasome (reviewed in [17–19]), and is associated with the progression of retinal pathologies including neovascular and atrophic AMD [20, 21]. Several studies have also indicated that IL-1β is secreted by microglia in photo-oxidative damage [22, 23], as well as in models of neovascular AMD [24], retinitis pigmentosa [25], and retinal detachment [26]. In this study, we tested the hypothesis that IL-1β promotes the up-regulation of chemokines in Müller cells and RPE, increasing outer-retinal macrophage accumulation and photoreceptor death, using a model of focal retinal degeneration. In this model, several inflammatory features observed in atrophic AMD are produced, including the expression of chemokines such as Ccl2, macrophage accumulation and outer retinal lesion development [27–29]. We find that inhibiting IL-1β, either via antibody neutralisation or targeted small interfering RNA (siRNA), suppresses the expression of RPE- and Müller cell-associated chemokines *Ccl2*, *Cxcl1*, and *Cxcl10*, reduces accumulation of macrophages in the outer retina, and mitigates photoreceptor death. We also find that IL-1β protein directly stimulates retinal chemokine up-regulation in vivo, and in cultured RPE and Müller cells. Targeting IL-1β as a therapeutic approach to reduce chemokine synthesis in the damaged retina may be beneficial in slowing the progression of retinal degenerations.

## Results

### *Expression of retinal* IL-1β *in relation to chemokine up-regulation and macrophage infiltration following photo-oxidative damage*

**Fig. 1:**
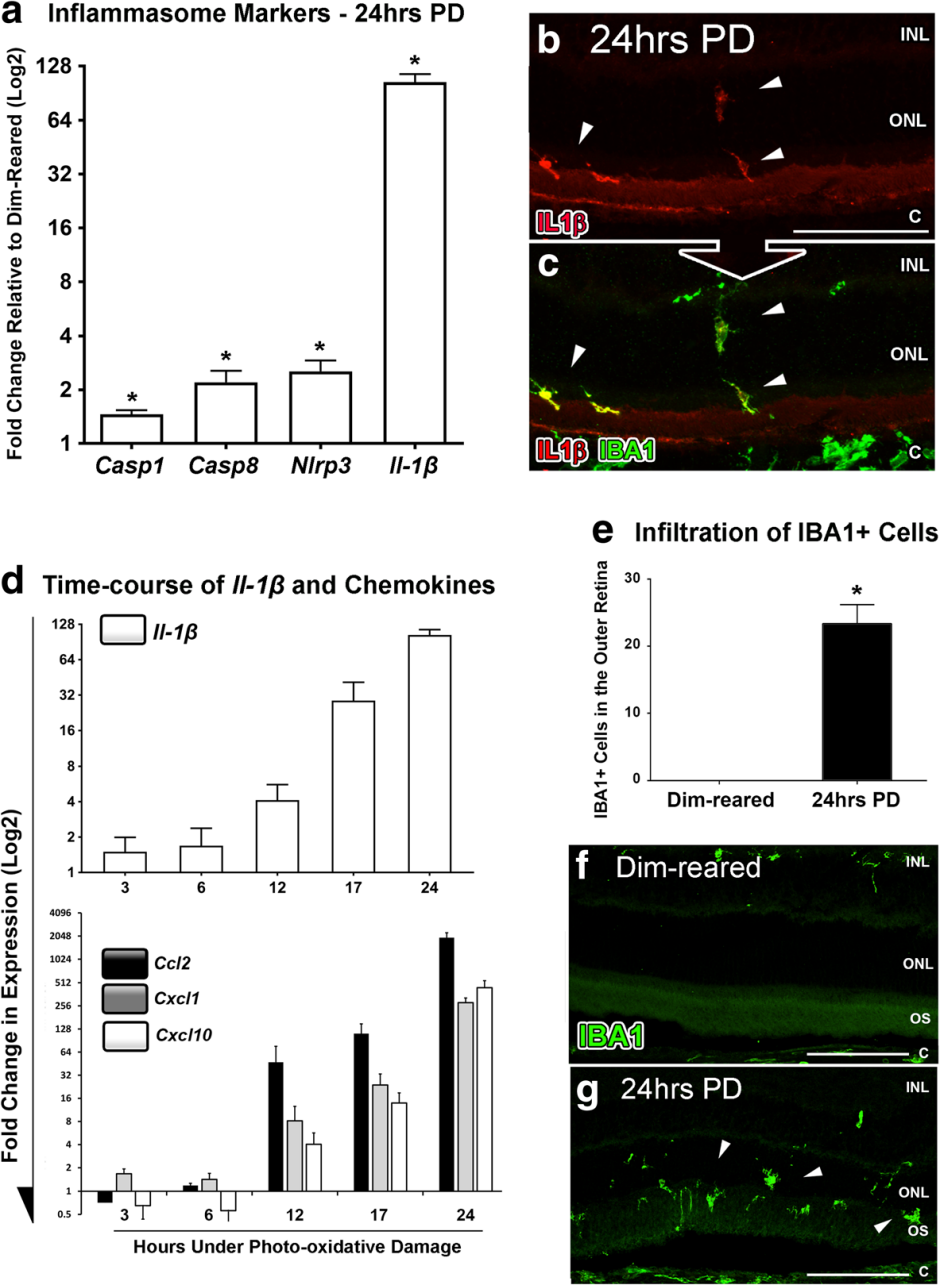
Temporal relation of IL-1β to chemokine expression and macrophage infiltration following photo-oxidative damage (PD). **a** After 24 h of light exposure, a number of inflammasome activation markers were significantly upregulated (*Casp1, Casp8, Nlrp3, P <* 0.05), in addition to *Il-1β* (*P <* 0.05). **b-c** Representative images show immunoreactivity for IL-1β (*red*) in the outer retina following 24 h of photo-oxidative damage, particularly in the ONL, subretinal space and RPE (B; arrows), and which co-localised to IBA1+ microglia (green) (C; arrows). **d** Up-regulation of IL-1β was documented over a 24 h time-course of photo-oxidative damage (*P <* 0.05), which was found to align closely with up-regulation of *Ccl2*, *Cxcl1* and *Cxcl10* over the same period (*P <* 0.05). **e** A large increase in the number of IBA1-immunoreactive macrophages was quantified within the outer retina (ONL and subretinal space) following photo-oxidative damage (*P <* 0.05) compared to dim-reared controls. **f**-**g** Representative images showcase the infiltration of IBA1-immunolabelled macrophages (*green*) within the ONL and subretinal space (arrows) in retinal sections following photo-oxidative damage (**g**), in contrast to the absence of these cell in sections from dim-reared retinas (**f**). **c**, choroid; INL, inner nuclear layer; ONL, outer nuclear layer; OS, outer segments. *N =* 4-6 per group. Asterisks denote a significant change, where *P <* 0.05. Scale bars equate to 50 μm

Retinal expression of *Il-1β* and genes associated with inflammasome assembly and activation (*Casp1*, *Casp8*, and *Nlrp3)* were assessed by qPCR following 24 h photo-oxidative damage (Fig. 1a). *Il-1β* was dramatically up-regulated after 24 h photo-oxidative damage, consistent with our prior reports [13], and in concert with expression of *Casp1*, *Casp8*, and *Nlrp3* (*P* < 0.05, Fig. 1a). Immunoreactivity for IL-1β was not detected in dim-reared control retinas, but after photo-oxidative damage IL-1β immunoreactivity was evident on some ramified IBA1+ microglia/macrophages in the ONL and subretinal space (Fig. 1b-c, yellow). IBA1+ cells located in the choroid did not exhibit IL-1β immunoreactivity (Fig. 1c).

Comparison of *Il-1β* expression with changes in retinal *Ccl2*, *Cxcl1* and *Cxcl10* (Fig. 1d) shows a correlation between *Il-1β* and chemokine expression. Over the 24 h time-course of photo-oxidative damage (3, 6, 12, 17, and 24 h), *Il-1β* expression was markedly upregulated after 6 h, and increasing *Il-1β* expression was associated with an upregulation of *Ccl2*, *Cxcl1*, and *Cxcl10*, with all markers reaching peak expression at 24 h (*P* < 0.05; one-way ANOVA). Consistent with previous reports [28, 29] we also observed incursions of IBA1+ macrophages into the ONL and subretinal space by 24 h of photo-oxidative damage (*P* < 0.05, Fig. 1e-g).

### *Effect of* IL-1β *suppression on photoreceptor death, macrophage accumulation, and chemokine expression*

**Fig. 2:**
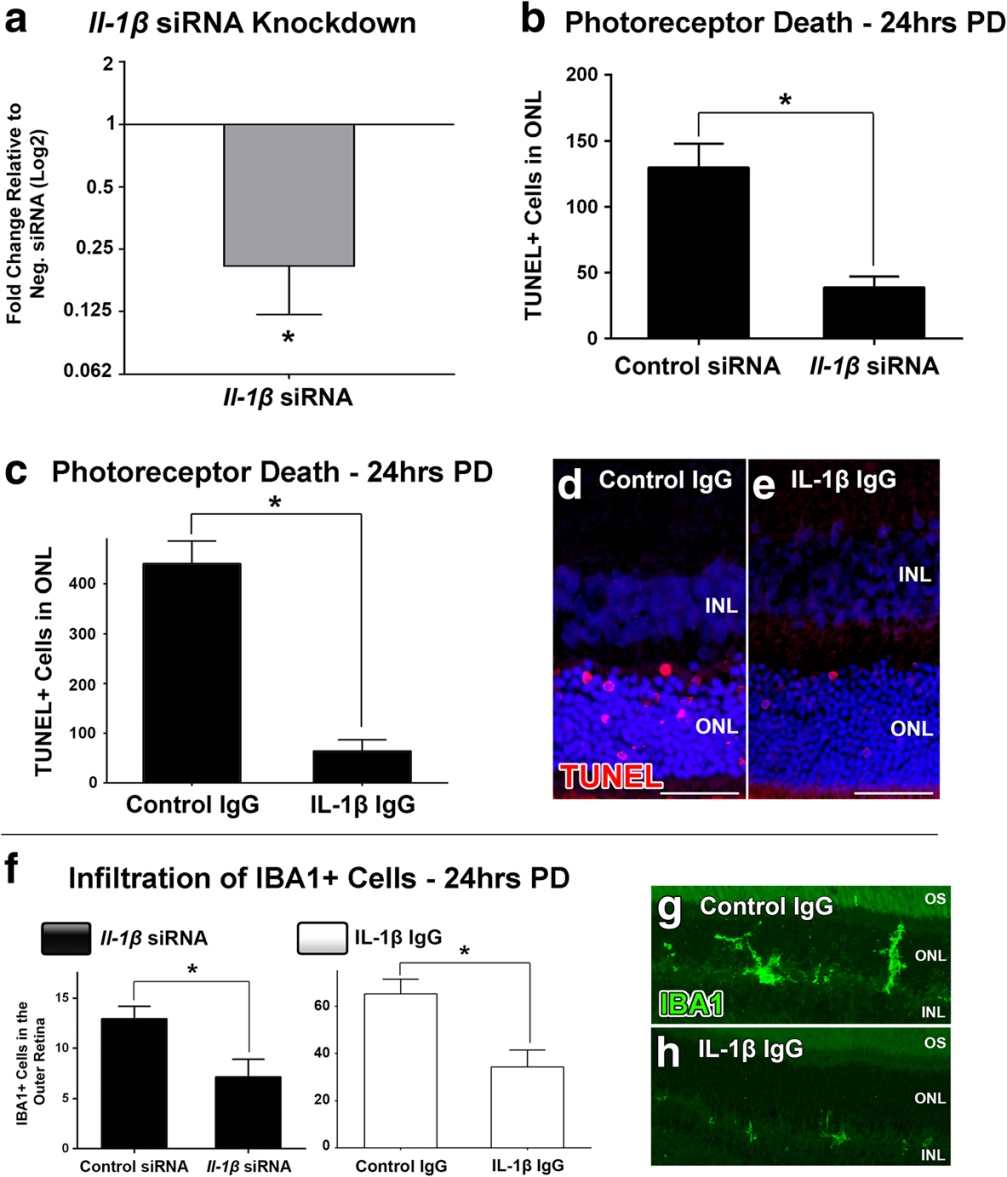
Effect of IL-1β inhibition on photoreceptor apoptosis and macrophage infiltration following photo-oxidative damage (PD). **a** A significant knockdown of *Il-1β* expression in the retina was achieved in retinas injected with an *Il-1β*-specific siRNA, compared to the negative control siRNA group (*P <* 0.05). **b** The number of TUNEL+ cells in the ONL was reduced in *Il-1β* siRNA-injected retinas after photo-oxidative damage, compared to controls (*P <* 0.05) **c** A reduction in the number of TUNEL+ cells in the ONL was also documented in animals that had been intravitreally injected with an IL-1β neutralising antibody prior to photo-oxidative damage, in comparison to an isotype control antibody (*P <* 0.05). **d**-**e** Representative images showcase a decrease in TUNEL+ profiles (*red*) in retinal section from the IL-1β neutralisation group (**e**), compared to a section from the isotype control group (**d**). **f** The infiltration of IBA1-immunolabelled macrophages into the outer retina (ONL and subretinal space) following photo-oxidative damage was significantly decreased in animals injected with either *Il-1β*-specific siRNA or an IL-1β-specific neutralising antibody, compared to their respective controls (*P <* 0.05). **g**-**h** Representative images of IBA1-immunolabelled macrophages demonstrate the reduction in IBA1+ cells in the outer retina in the IL-1β antibody neutralisation retinas compared to controls (*green*). INL, inner nuclear layer; ONL, outer nuclear layer. *N =* 4-5 per group. Asterisks denote a significant change, where *P <* 0.05. Scale bars equate to 25 μm

**Fig. 3:**
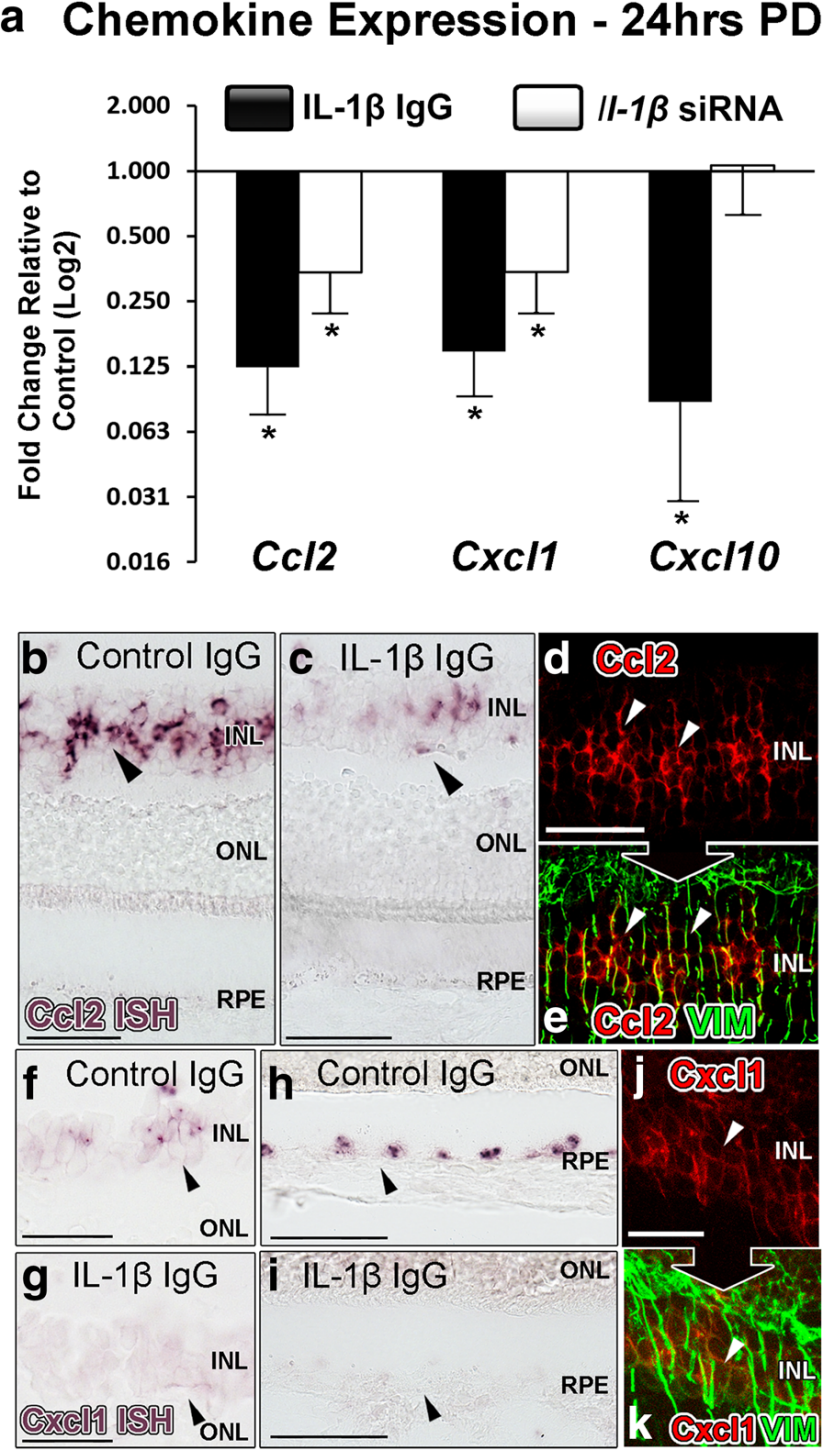
Effect of IL-1β inhibition on chemokine expression following photo-oxidative damage (PD). **a** In animals that were intravitreally injected with *Il-1β*-specific siRNA, there was significantly lower expression of *Ccl2* and *Cxcl1* compared to control siRNA after photo-oxidative damage (*P <* 0.05). In the group injected with IL-1β neutralising antibody however, the expression of *Ccl2*, *Cxcl1* and *Cxcl10* were all significantly down-regulated in comparison to the isotype control group (*P <* 0.05). **b**-**k** *In situ* hybridisation was used to examine the localisation of *Ccl2* and *Cxcl1* mRNA transcripts following IL-1β inhibition and photo-oxidative damage, as shown in representative images. Staining for *Ccl2* mRNA (*purple/red*) was decreased in IL-1β antibody-injected retinas compared to control IgG retinas (**b**-**c**; arrows), and was co-localised to vimentin–immunoreactive Müller cells (*green*) (D-E; arrows). Staining for *Cxcl1* mRNA was observed within INL (**f**-**g**) and RPE layers (**h**-**i**), which was decreased in the IL-1β neutralising antibody group. The INL staining correlated with Müller cell processes that were immunolabelled with vimentin (**j**-**k**). INL, inner nuclear layer; ONL, outer nuclear layer; OS, outer segments. *N =* 5 per group. Asterisks denote a significant change, where *P <* 0.05

Inhibition of IL-1β and its effect on photo-oxidative retinal damage was ascertained using both siRNA and antibody neutralisation approaches (Figs. 2 and 3). Intravitreal injection of the *Il-1β*-specific siRNA induced a 1.8 fold reduction in the expression of retinal *Il-1β* at 24 h photo-oxidative damage, compared to negative control siRNA (*P* < 0.05, Fig. 2a). Animals injected with *Il-1β* siRNA had ~60% fewer TUNEL+ photoreceptors 24 h post-exposure to photo-oxidative damage compared to controls (*P* < 0.05, Fig. 2b). In experiments where IL-1β was neutralised using the antibody, injected intravitreally prior to photo-oxidative damage, there was an 80% reduction in the number of TUNEL+ photoreceptors in the retina, compared to those injected with an isotype antibody control (*P* < 0.05, Fig. 2c-e). Counts of IBA1+ macrophages confirm that inhibition of IL-1β with either siRNA or neutralising antibody reduces the number of IBA1+ macrophages in the outer retina (ONL and subretinal space), compared with the respective controls (*P* < 0.05, Fig. 2f-h).

We then sought to determine the effect of IL-1β suppression on the retinal expression of chemokines *Ccl2, Cxcl1,* and *Cxcl10* (Fig. 3a). In both modes of IL-1β inhibition, there was a significant reduction in the expression of *Ccl2* and *Cxcl1* compared to controls (*P* < 0.05, Fig. 3a). While *Il-1β*-specific siRNA did not modify expression of *Cxcl10*, antibody neutralisation of IL-1β was effective in reducing *Cxcl10* expression (*P* < 0.05). By use of *in situ* hybridisation, we also confirmed that *Ccl2* mRNA was present in vimentin-immunoreactive Müller cell processes after 24 h photo-oxidative damage (Fig. 3d-e; arrows), and that *Ccl2* mRNA labelling was reduced in IL-1β-inhibited retinas compared to controls (Fig. 3b-c; arrows). *Ccl2* mRNA was not detected in RPE cells (Fig. 3b-c), consistent with our previous findings [13, 28]. We detected *Cxcl1* mRNA labelling in the INL (Fig. 3f; arrows) and RPE layer (Fig. 3 h; arrows) after photo-oxidative damage, which was reduced in retinas where IL-1β had been inhibited via neutralising antibody (Fig. 3 g, i). INL staining for *Cxcl1* mRNA correlated with vimentin-immunoreactive Müller cells (Fig. 3 j-k; arrows), consistent with our previous report [13].

### *Effect of* IL-1β *stimulation on chemokine expression in Müller and RPE cell cultures*

**Fig 4:**
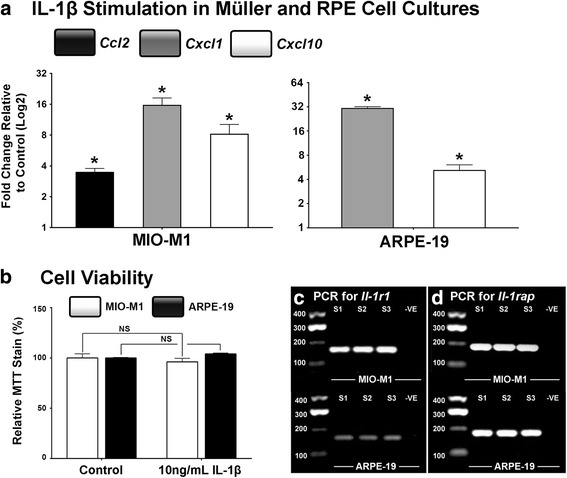
Chemokine expression in RPE and Müller cell cultures stimulated with IL-1β. **a** MIO-M1 and ARPE-19 cells were incubated with IL-1β protein for 12 h, after which MIO-M1 cultures were found to up-regulate expression of *Ccl2, Cxcl1* and *Cxcl10* (*P <* 0.05), while ARPE-19 cells had significantly increased expression of *Cxcl1* and *Cxcl10* (*P <* 0.05), compared to unstimulated controls. **b** MTT assays were conducted on MIO-M1 and ARPE-19 cultures, which showed no difference in cell viability between IL-1β and control groups. **c**-**d** Representative images of *Il-1r1* and *Il-rap* PCR products indicate that MIO-M1 and ARPE-19 express the receptor genes necessary for *Il-1β* signal transduction. *N =* 5 per group. Asterisks denote a significant change, where *P <* 0.05

The capacity for IL-1β to stimulate chemokine up-regulation in Müller cells and RPE cells was assessed in MIO-M1 and ARPE-19 cell cultures, respectively (Fig. 4). Both cultures were incubated with 10 ng/mL IL-1β protein for 12 h, at which point we observed dramatic increases in the expression of *Ccl2*, *Cxcl1* and *Cxcl10* in MIO-M1 cells compared to unstimulated control wells (*P* < 0.05, Fig. 4a). To discount the possibility that the IL-1β-induced up-regulation of chemokines was a result of cell stress/death, we conducted an MTT assay on the cultures after IL-1β stimulation (Fig. 4b). We determined that both cultures exhibited no reduction in viability as a result of IL-1β stimulation compared to controls (*P >* 0.05). We also found that both MIO-M1 and ARPE-19 cells express *Il-1r1* and *Il-1rap* receptor genes necessary for IL-1β signal transduction (Fig. 4c-d).

### *Changes in chemokine expression and macrophage infiltration following intravitreal delivery of* IL-1β *protein*

**Fig. 5:**
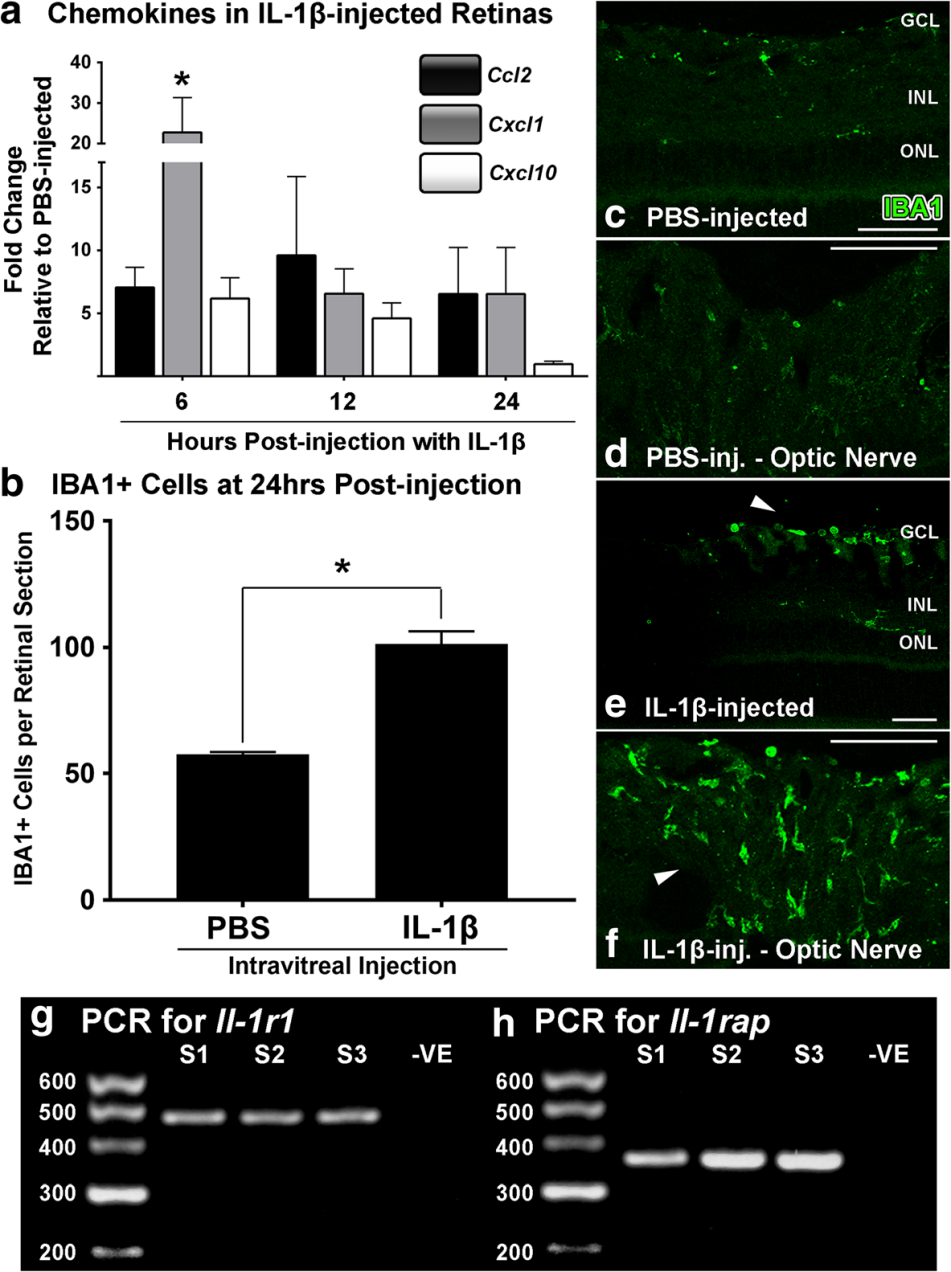
Change in retinal chemokine expression and macrophage infiltration in retinas following intravitreal injection of IL-1β protein. **a** Injection of IL-1β protein increased the expression of *Ccl2*, *Cxcl1* and *Cxcl10* in retinas over a time-course of 6, 12, and 24 h post-injection, compared to PBS-injected controls (*P <* 0.05). **b** There was a significant increase in the number of IBA1+ cells throughout retinal sections in the IL-1β group at 24 h post-injection, compared to the PBS control group (*P <* 0.05). **c**-**f** Representative images of IBA1-immunolabelled retinal sections show increased clusters of IBA1+ macrophages amongst the GCL and optic nerve head of the IL-1β-injected group (**e**-**f**; arrows) compared to PBS controls (**c**-**d**). **g**-**h** Representative images show positive expression of PCR products for *Il-1r1* and *Il-rap* in control rat retinas. GCL, ganglion cell layer; INL, inner nuclear layer; ONL, outer nuclear layer. *N =* 3-6 per group. Asterisks denote a significant change, where *P <* 0.05. Scale bars equate to 50 μm

Finally, we investigated the effect of IL-1β protein administered intravitreally on the expression of chemokines and the accumulation of macrophages over a 24 h period (Fig. 5). The data show a broad up-regulation of retinal *Ccl2*, *Cxcl1*, and *Cxcl10* (Fig. 5a), up to 24 h post-injection compared to PBS-injected controls. This was particularly evident for *Cxcl1*, which by 6 h-post-injection increased ~22 fold (*P <* 0.05), although was somewhat reduced by 12 and 24 h. The localisation and number of IBA1+ macrophages at 24 h after the injection of IL-1β protein (Fig. 5b-f) demonstrated a significant increase in the total number of retinal macrophages present compared to the PBS-control group (*P <* 0.05; Fig. 5b). IBA1+ cells were observed predominantly in the GCL and optic nerve head (Fig. 5e-f; arrows), and comprised a population with a rounded (activated) rather than ramified (resting) morphology. PCR conducted on isolates of control rat retinal tissue confirmed the presence of *Il-1r1* and *Il-1rap* genes (Fig. 5 g-h).

## Discussion

These findings describe for the first time a key role for IL-1β in mediating the accumulation of outer-retinal macrophages by modulating the expression of chemokines by Müller cells and RPE. First, we demonstrate that *Il-1β* is up-regulated in concert with the Müller and RPE cell-expressed chemokines (*Ccl2*, *Cxcl1* and *Cxcl10*), and with the influx of macrophages into the outer retina following photo-oxidative damage. Second, we showed that therapeutic suppression of retinal IL-1β using either siRNA or antibody neutralisation curtails chemokine expression, accumulation of outer-retinal macrophages and photoreceptor degeneration. Finally, we show that Müller and RPE cell cultures up-regulate *Ccl2*, *Cxcl1* and *Cxcl10* in response to IL-1β stimulation, and that intravitreal delivery of IL-1β induces up-regulation of these same chemokines in the retina, coinciding with increases in the population of retinal macrophages. The data from this study supports the use of IL-1β inhibition strategies as a therapeutic approach to reduce chemokine synthesis, and subsequent macrophage accumulation and photoreceptor death in retinal degenerations.

Previous studies using injections of recombinant IL-1ra, an endogenous antagonist for IL-1r1, have suggested a role for IL-1β in propagating retinal degeneration, using models of photo-oxidative damage in Cx3cr1-deficient mice [23, 30], laser-induced CNV [24], and retinitis pigmentosa [25]. However, a short-coming of that approach is that IL-1ra does not suppress IL-1β signalling specifically, as the inflammatory cytokine IL-1α also competes for binding and is equally as effective in activating Il-1r1 as IL-1β [19, 31, 32]. In this study, we have used *Il-1β*-specific siRNA, as well as IL-1β antibody inhibition methodologies to target IL-1β directly, circumventing any possibility of off-target inhibition of IL-1α-mediated signalling. Our data clearly show that decreased IL-1β correlates with decreased chemokine production, and increased photoreceptor survivability. Excluding the effect of IL-1α signalling is an important consideration, as IL-1α released from dying cells promotes sterile inflammation and leukocyte recruitment [32, 33], and ablation of IL-1α alleviates inflammation in myocardial infarction [34]. Moreover, our previous microarray analysis (gene expression omnibus GSE22818) indicates that IL-1α is up-regulated following photo-oxidative damage [29].

Our previous investigations have indicated that RPE and Müller cells are potentiators of *Ccl-* and *Cxcl-* expression during photo-oxidative damage [13, 15, 28]. Several of these chemokines, including CCL2, CXCL1 and CXCL10 are involved in leukocyte recruitment in CNS diseases and in retinal degeneration [7, 12, 13, 28]. The significance of the expression of such chemokines is underscored by our finding that the broad spectrum chemokine inhibitor NR58-3.14.3 – a suppressor of Ccl- and Cxcl- signalling – ameliorates macrophage recruitment and photoreceptor degeneration resulting from photo-oxidative damage [14]. The involvement of IL-1β signalling in chemokine expression shown in the current study is consistent with its known influence outside the retina, including its ability to induce CCL2 in pancreatic β-cells [35, 36], and spur up-regulation of CXCL1 in intestinal tissue during infection with *Clostridium difficile* [37]. Precisely how IL-1β induces the up-regulation of chemokines in RPE and Müller cells is uncertain, though it has been demonstrated in pancreatic β-cells that IL-1β signalling mediates nuclear localisation of the transcription factor NF-κB, which then promote the expression of chemokines such as CCL2 [38].

While the data generated in these investigations was generally consistent between the IL-1β inhibition strategies employed, it is noted that expression of *Cxcl10* was not inhibited by *Il-1β* siRNA, in contrast to the findings using antibody neutralisation. This difference may be due to delayed efficacy of the siRNA resulting from the time required for adequate transfection and mRNA suppression. Consistent with this idea, comparison of the data indicate that the neutralising antibody had a more potent effect on *Ccl2* and *Cxcl1* expression compared with siRNA. However, given the reduction in macrophage infiltration identified in both treatment groups (siRNA and antibody), despite the discordant suppression of *Cxcl10*, it is possible that *Cxcl10* does not play a crucial role in macrophage recruitment in the retina compared to *Ccl2* and *Cxcl1*. While indeed plausible, other studies have shown that CXCL10 specifically elicits macrophage recruitment in experimental nonalcoholic steatohepatitis [39], and is also implicated in macrophage infiltration in kidney during puromycin aminonucleoside nephrosis [40].

Though our investigation focused on the effect of IL-1β on expression of RPE/Müller cell-associated chemokines and macrophage recruitment, the potential contribution of other leukocyte populations should not be overlooked. Peripheral neutrophils and T-cells, as well as macrophages, express the receptors for CXCL1 and CXCL10 [41–44]. These leukocyte populations are poorly characterised in sterile retinal inflammation, although are understood to comprise a small proportion amongst the predominantly macrophage-led response in AMD and models such as photo-oxidative damage [45, 46]. Nonetheless, the contribution of these cell types to pathology in sterile retinal inflammation is unclear, and identifies neutrophils and T-cells as candidates for investigation in future studies.

## Conclusions

Our study identifies a key role for IL-1β in orchestrating the infiltration of macrophages to the outer retina, by inducing the up-regulation of chemokines in RPE and Müller cells following retinal damage. Moreover, we confirm the potential of specific IL-1β inhibitors in dampening inflammation and ameliorating photoreceptor degeneration. Consequently, their application may have value in the treatment of retinal dystrophies in which chemokine expression and subretinal macrophage accumulation are implicated, such as AMD.

## Methods

### Animals and photo-oxidative damage

All experiments were conducted in accordance with the ARVO Statement for Use of Animals in Ophthalmic and Vision Research, and had approval from the Australian National University (ANU) Animal Experimentation Ethics Committee (Ethics ID: A2014/56). Adult Sprague–Dawley (SD) albino rats aged were used for all experiments. Animals were born and reared under dim light conditions (5 lux) prior to photo-oxidative damage. For the photo-oxidative damage paradigm, animals were placed into transparent Perspex open-top cages under a light source (COLD F2, 2x36W, IHF, Thorn Lighting, Australia) at 1000 lux for either 3, 6, 12, 17, or 24 h (hrs), with access to food and water *ad libitum*. Following light exposure, animals were immediately euthanized using an overdose of barbiturate via an intraperitoneal injection (Valabarb; Virbac, NSW, Australia). For each animal, eyes were processed for either cryosectioning or RNA extraction, according to protocols detailed in our previous publications [29].

### Intravitreal injections

Intravitreal injections were performed as described in detail previously [47], wherein animals were anaesthetised using an intraperitoneal injection of ketamine (100 mg/kg; Troy Laboratories, NSW, Australia) and xylazil (12 mg/kg; Troy Laboratories). Injections consisted of either siRNA- or antibody-based IL-1β inhibitors, or IL-1β protein.

A neutralising antibody to IL-1β (Cat# AF-501-NA, R&D Systems, Minneapolis, MN) was administered intravitreally to rats immediately prior to photo-oxidative damage. A 3 μL solution containing either anti-IL-1β or an isotype control antibody was injected into individual animals, which equated to a delivery of 0.6 μg of antibody per eye. After intravitreal injections, the animals were immediately transferred to individual cages designed to allow light to enter unimpeded. Animals were exposed to photo-oxidative damage for 24 h, during which corneal hydration was maintained though application of a synthetic tear gel (GenTeal Gel; Novartis, NSW, Australia) until the animals awoke.

RNA-interference (RNAi) was conducted using *Il-1β*-specific siRNA (Cat# s127941; Thermo Fisher Scientific, Waltham, MA, USA), while a scrambled negative siRNA (Cat# 12935300, Stealth RNAi Med GC; Thermo Fisher Scientific) served as a control, which were encapsulated using a cationic liposome-based formulation (Invivofectamine 3.0 Reagent; Thermo Fisher Scientific) according to the manufacturer’s instructions. To purify and concentrate the siRNA formulation, the samples were centrifuged at 4000 g through an Amicon Ultra-4 Centrifugal Filter Unit (Merck Millipore, MA, USA). The final concentration of the encapsulated siRNA formulation was 1 μg/μl in endotoxin-free 0.1 M PBS. For injection, animals were anaesthetised in the same fashion as the antibody neutralisation series. 3 μl of either *Il-1β* or negative siRNA was then intravitreally delivered to both eyes of each animal, which equated to a final dosage of 3ug siRNA per eye. Animals were then exposed to 24 h photo-oxidative damage under the same parameters as the antibody neutralisation cohort.

IL-1β protein was administered to rats following the same intravitreal methodology as described in the inhibition experiments. Recombinant IL-1β protein (Cat# AF-501-RL-010, R&D Systems) was injected at final concentration of 10 ng per eye, as established in a previous study [48]; injections with only the PBS vehicle served as controls. Animals were euthanized at 3, 6, or 12 days post-injection, with eyes then processed as described in the previous section.

### RPE and Müller cell cultures

Immortalised human cell lines MIO-M1 (Müller 1 Moorefields Institute of Ophthalmology; Dr A. Limb, Institute of Ophthalmology, University College London) and ARPE-19 (ATCC CRL-2302, American Tissue Culture Collection, Manassas, VA) were used to study responses to IL-1β stimulation. Cell lines were authenticated by CellBank, Australia. The cells were grown in Dulbecco's Modified Eagle Medium (DMEM; Life Technologies, Carlsbad, CA) supplemented with 10% fetal bovine serum (FBS; Life Technologies) and 3 mM L-glutamine (Life Technologies), in a humidified atmosphere consisting of 95% air and 5% CO_2_ at 37 °C. The cells were passaged by trypsinization every 3 to 4 days.

The MIO-M1 and ARPE-19 lines were incubated in IL-1β protein to assess its effect on their expression of chemokines. Cells were first seeded to a density of 1 × 10^5^ cells on 24-well plates and left to recover for at least 48 h. 24 h prior to experimentation, the cells were placed in serum-deficient DMEM containing 1% FBS. IL-1β protein (Cat# 201-LB, R&D Systems) was then added to the culture medium at a concentrations of 10 ng/mL, as per previous literature [49, 50], and then incubated for 12 h. The cells were then harvested for either RNA extraction and PCR, or an MTT assay to verify cell viability. RNA was extracted from each sample well using a retinal RNA extraction protocol that we have established previously [28], with slight modifications for cell culture. The MTT assay was performed with a kit supplied by Roche (Cell Proliferation Kit I, Roche Applied Science, Penzberg, Germany) following the supplied instructions. Following IL-1β stimulation, MTT reagent was added to the sample wells and left to incubate for 4 h, after which a solution of 0.04 M HCL in isopropanol was then added to each well to dissolve the resulting formazan crystals. The absorbance for each sample was then read at 570 nm on a TECAN Infinite 200 PRO (TECAN Seestrasse, Männedorf, Switzerland), and quantified as a percentage relative to unstimulated culture samples.

### TUNEL and immunohistochemistry

Retinal cryosections were stained for apoptotic cells using a terminal deoxynucleotidyl transferase dUTP nick end labelling (TUNEL) kit (Roche Applied Science) and following our previous methodology [51, 52]. To quantify photoreceptor cell death, TUNEL+ cells in the ONL were counted throughout the full length of each section cut in the parasagittal plane (supero-inferior). For each animal, technical duplicates were counted, and these counts were averaged for each experimental group.

Immunohistochemistry was performed on retinal cryosections according to previously described protocols, with minor modifications [13]. A list of primary antibodies used is provided in Table 1. Fluorescence in sections was captured using a laser-scanning A1^+^ confocal microscope (Nikon, Tokyo, Japan). Images were processed using Photoshop CS6 software (Adobe Systems, CA, USA). Immunolabelled IBA1+ microglia/macrophages were quantified across the full length of each section in the parasagittal plane (supero-inferior). The number of IBA1+ microglia/macrophages in the outer retina was quantified by counting the IBA1+ cells in the ONL and subretinal space.

**Table 1:** Primary antibodies used for immunohistochemistry

Primary antibody	Dilution	Source
Rabbit α-IBA1 (ionized binding calcium adaptor molecule 1)	1:500	#019–19741, Wako Pure Chemical Industries, Osaka, Japan
Goat α-IL-1β	1:500	#AF501, R&D Systems, Minneapolis, MN, USA
Mouse α-Vimentin	1:100	#V6630, Sigma-Aldrich, St. Louis, MO, USA

### Polymerase chain reaction (PCR)

In preparation for quantitative real-time PCR (qPCR) and standard PCR, the RNA from retina or cell culture samples was synthesised into cDNA using a Tetro first-strand cDNA Synthesis Kit (Bioline Reagents, London, UK), as described in our previous investigation [13].

qPCR was performed on cDNA samples using Taqman hydrolysis probes (Table 2; Thermo Fisher Scientific), which were applied according to the manufacturer’s instructions with the Taqman Gene Expression Master Mix system (Thermo Fisher Scientific). The qPCR reactions were run on a QuantStudio Flex 12 K instrument (Thermo Fisher Scientific). The resultant data were analysed according to the comparative cycle threshold (C_t_) method (ΔΔC_t_), which was normalised to the expression of both *Gapdh* and *Actb* reference genes, as established in our previous analyses [28, 53].

**Table 2:** Taqman hydrolysis probes used for qPCR

Gene symbol	Gene name	Catalog number	Entrez gene ID
*Actb*	Actin, beta	Rn00667869_m1	81822
*Casp1*	Caspase 1	Rn00562724_m1	25166
*Casp8*	Caspase 8	Rn00574069_m1	64044
*Ccl2*	Chemokine (C-C) motif ligand 2	Rn01456716_g1	24770
*Cxcl1*	Chemokine (C-X-C) motif ligand 1	Rn00578225_m1	81503
*Cxcl10*	Chemokine (C-X-C) motif ligand 10	Rn01413889_g1	245920
*Gapdh*	Glyceraldehyde-3-phosphate dehydrogenase	Rn99999916_s1	24383
*Il-1β*	Interleukin 1β	Rn00580432_m1	24494
*Nlrp3*	Nucleotide-binding domain, leucine-rich-containing family, pyrin domain-containing-3	Rn04244620_m1	287362

Standard PCR was conducted from cDNA synthesised from cells cultures or retinal homogenates, using primers specific to *Il-1β* receptor-related genes in human: *Il-1r1* (F: 5’ ATCGTGATGAATGTGGCTGA 3’; R: 5’ TCTCATTAGCTGGGCTCACA 3’), *Il-1rap* (F: 5’ CGTTTCATCTCACCAGGACTC 3’; R: 5’ CCAAACCTCATTGCGAGAAT 3’), or rat: *Il-1r1* (F: 5’ ACATTCGCAGCTGTCCTCTT 3’; R: 5’ TGTGCTCTTCAGCCACATTC 3’), *Il-1rap* (F: 5’ TCATACCGCCAAGGTACACA 3’; R: 5’ GGGCTCAGGACAACAATCAT 3’). The primers designed using the Primer3 web-based design program [54]; both transverse an intron splice site to avoid genomic amplification. The PCR was performed using MyTaq DNA Polymerase (Bioline) as per the manufacturer’s instructions, and the presence and specificity of the PCR product were inferred by gel electrophoresis.

### *In situ* hybridisation

*Ccl2* and *Cxcl1* were cloned from PCR products (550-bp and 504-bp amplicons respectively) using cDNA synthesised from retinal RNA (as described above). Digoxigenin (DIG)-labelled riboprobes were then prepared as described in our previous publication [13]. *In situ* hybridisation on retinal cryosections was carried out according to our established methodology [55]; briefly, each riboprobe was hybridised to sections overnight at 55 °C, then was washed in decreasing concentrations of saline sodium citrate (pH 7.4) at 60 °C. The bound probe was visualised with either NBT/BCIP or HNPP/Fast-Red (Roche Applied Science).

### Quantitative and statistical analysis

Graphing and statistical analysis for this study was performed using Prism 7 (GraphPad Software, CA, USA). Statistical analysis was conducted using a Students *t*-test for instances of single comparisons. For assessing trends or multiple comparisons over protracted time-courses, a Kruskal–Wallis one-way analysis of variance (ANOVA) with Dunn’s multiple comparison post-test was applied, as per our previous investigation [13]. A *P* value of < 0.05 was considered statistically significant.

## Abbreviations

AMD: Age-related macular degeneration
ANOVA: Analysis of variance
ANU: Australian National University
ARPE-19: ATCC CRL-2302 (human RPE cell line)
ARVO: Association for Research in Vision and Ophthalmology
BCIP: 5-bromo-4-chloro-3-indolyl phosphate
C: Choroid
CASP-1: Caspase 1
CASP-8: Caspase 8
CCL2: Chemokine (C-C) motif ligand 2
CCR2: Chemokine (C-C) motif receptor 2
cDNA: Complementary DNA
CNV: Choroidal neovascularisation
C_t_: Cycle threshold
CX3CR1: Chemokine (C-X3-C) motif receptor 1
CXCL1: Chemokine (C-X-C) motif ligand 1
CXCL10: Chemokine (C-X-C) motif ligand 10
DIG: Digoxigenin
DMEM: Dulbecco’s modified eagle’s medium
DNA: Deoxyribonucleic acid
dUTP: 2’-deoxyuridine 5’-triphosphate
FBS: Fetal bovine serum
GCL: Ganglion cell layer
HNPP: 2-hydroxy-3-naphthoic acid-2’-phenylanilide phosphate
hr(s): Hour(s)
IBA-1: Ionised calcium binding adaptor molecule 1
IgG: Immunoglobulin G
IL-1r1: Interleukin 1 receptor type I
IL-1ra: Interleukin 1 receptor antagonist
IL-1rap: Interleukin 1 receptor accessory protein
IL-1α: Interleukin 1α
IL-1β: Interleukin 1β
IL-6: Interleukin 6
INL: Inner nuclear layer
ISH: *in situ* hybridisation
LPS: Lipopolysaccharide
MIO-M1: Müller 1 Moorefields institute of ophthalmology (human Müller cell line)
mRNA: Messenger RNA
MTT: 3-(4,5-dimethylthiazol-2-yl)-2,5-diphenyltetrazolium bromide
NBT: Nitro blue tetrazolium
NF-κB: Nuclear factor kappa-light-chain-enhancer of activated B cells
NLRP3: Nucleotide-binding domain, leucine-rich-containing family, pyrin domain-containing-3
ONL: Outer nuclear layer
OS: Outer segments
PBS: Phosphate-buffered saline
PCR: Polymerase chain reaction
PD: Photo-oxidative damage
qPCR: Quantitative real-time PCR
RNA: Ribonucleic acid
RNAi: RNA interference
RPE: Retinal pigment epithelium
SD: Sprague–Dawley
siRNA: Small interfering RNA
TUNEL: Terminal deoxynucleotidyl transferase dUTP nick end labelling
VIM: Vimentin
ΔΔC_t_: Comparative cycle threshold
